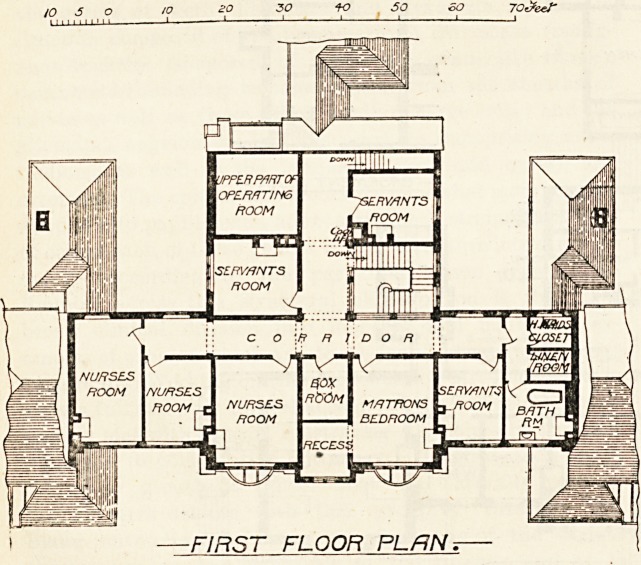# Woburn Cottage Hospital

**Published:** 1901-08-24

**Authors:** 


### THE INSTITUTIONAL WORKSHOP.

### WOBURN COTTAGE HOSPITAL.

THE district of Woburn owes this hospital, which is now being erected, entirely to the liberality of the Duke of Bedford, whose example in this matter might well be followed in many other parts of the country. The hospital is situated on an open site, and it faces Woburn Park. For convenience of description the ground plan may be divided into four parts—a centre, two wings, and an administrative or domestic portion.

The main entrance is, of course, in the centre of the south front, and the corridor leading from it joins the east-and-west corridor at right angles. On the right the dispensary take up the space occupied by the dining-room and dayroom in the east wing.

To thoroughly cross-ventilate a ward adapted for one bed only is a problem not easy of solution, and it has been effected here by placing a fanlight over the door of the small ward, which, being opposite the large window of the corridor, will materially help to keep the air of the room pure. The main ward is attached by its side to the corridor, instead of by its end. This arrangement we look upon as a mistake, and, as shown on the plan, two of the beds are placed against a wall having no windows. A nurses' kitchen and housekeeper's room are placed north of the east-and-west corridor, and so, on entering is the dining-room, which is well-proportioned and is sufficiently lighted by a circular window occupying almost the entire end of the room. Next to this are the dining-room and dayroom and a ward for one bed, and then one of the main wards for five beds. A verandah runs along two sides of this room, and there is a glass bay or sun-room at the corner, the bay being reached either from the verandah or from the ward. These adjuncts are both useful and ornamental. The sanitary block is placed to the north of the corridor of communication, where it will be but little in evidence. It contains bathroom, closet and sink, and well-ventilated cupboards are provided for the patients' clothes. The entrance to this block is correctly placed close to that of the main ward, and opposite that of the small ward, and a cross-ventilated, well-lighted passage cuts it off from the rest of the building. The west wing is similar, excepting that the matron's sitting-room and very properly, is the operation-room. This room has a north window of large size, and, what is of great importance, it has a roof-light in addition. This room is elaborately fitted up. To have left it otherwise would have been a mistake, more especially as the room may possibly be used by any surgeon in the county, a concession which is an index of enlightened policy. It seems a pity that the nurses' kitchen could not have been used as an anteroom to the operation-room. By this arrangement the doors opening to the north corridor could have been blocked and a new door made direct into the east-and-west corridor; but we confess we do not see any other suitable place for the nurses' kitchen than where it is; but the plan might have been modified a little.

The general kitchen, pantry, scullery, and larder are placed at the end of the north corridor, and these offices form a block by themselves, cut off from the main by a cross-ventilated passage. The staircase is close to this passage, and the first floor contains matron's bedroom, nurses' rooms, servants' rooms, bathroom, housemaid's closet and linen-room, and extra storerooms have been placed in the basement. We do not notice any water-closet at all on the first floor, but there is one at the foot of the staircase.

There is a very important annexe provided, in the shape of a small building where one or two patients could be isolated. This building contains sitting-room, kitchen, ward, and nurse's bedroom.

The mortuary is also entirely isolated.

The hospital will be warmed by open fireplaces; but hot-water pipes and radiators will be used as adjuncts. The ward floors are of polished teak, and the walls which have a dado of tiles, are finished above the dado by enamelled paint to render them non-absorbent and easily cleaned. The corners of the rooms are rounded off, and will not act as receptacles for dust.

The elevations are of rough-cast cement and English oak timber work, the oak being built in solid, as was the

—FIRST FLOOR PLAN.—

method in olden days, and not stuck on in strips, as is too often the practice in modern times. The roofs are covered with red tiles.

The sketch plans for the hospital were prepared by the Duchess of Bedford, and when it is stated that the drawings were worked out in detail by Mr. H. Percy Adams, it is tantamount to saying that much care has been exercised over them, and that the best possible materials have been used.

The cost of the hospital is not given. It would have been interesting to have it; but as it is entirely a private matter, the information can hardly be expected.

## Figures and Tables

**Figure f1:**
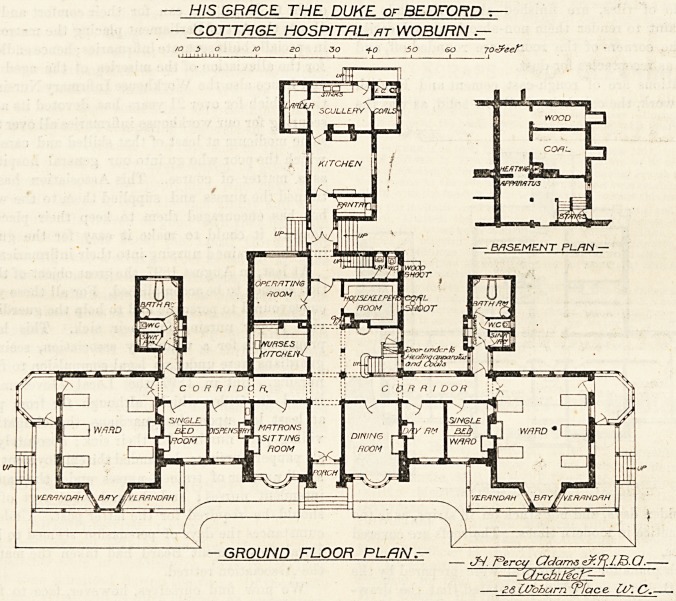


**Figure f2:**